# Characterization and potential of periosteum-derived cells: an overview

**DOI:** 10.3389/fmed.2023.1235992

**Published:** 2023-07-24

**Authors:** Rongkai Cao, Beibei Chen, Kun Song, Fang Guo, Haoxin Pan, Yujie Cao

**Affiliations:** ^1^Stomatological Hospital and Dental School of Tongji University, Shanghai, China; ^2^Department of Stomatology, The First Affiliated Hospital of Fujian Medical University, Fuzhou, China

**Keywords:** periosteum-derived cells, isolation, characterization, osteogenic, chondrogenic, adipogenic

## Abstract

As a thin fibrous layer covering the bone surface, the periosteum plays a significant role in bone physiology during growth, development and remodeling. Over the past several decades, the periosteum has received considerable scientific attention as a source of mesenchymal stem cells (MSCs). Periosteum-derived cells (PDCs) have emerged as a promising strategy for tissue engineering due to their chondrogenic, osteogenic and adipogenic differentiation capacities. Starting from the history of PDCs, the present review provides an overview of their characterization and the procedures used for their isolation. This study also summarizes the chondrogenic, osteogenic, and adipogenic abilities of PDCs, serving as a reference about their potential therapeutic applications in various clinical scenarios, with particular emphasis on the comparison with other common sources of MSCs. As techniques continue to develop, a comprehensive analysis of the characterization and regulation of PDCs can be conducted, further demonstrating their role in tissue engineering. PDCs present promising potentials in terms of their osteogenic, chondrogenic, and adipogenic capacities. Further studies should focus on exploring their utility under multiple clinical scenarios to confirm their comparative benefit over other commonly used sources of MSCs.

## Introduction

The periosteum, a thin fibrous layer covering the bone surface, consists of an inner cambium layer and an outer fibrous layer, both of which hold significant potential in tissue engineering. In addition, the periosteum is of great significance in bone physiology during growth, development, and remodeling. The osteogenic potential of periosteum was proposed firstly by Duhamel in 1742, followed by Ollier who confirmed that new bone formation could be induced through transplanted periosteum a century later (1). Extensive studies have examined the crucial influence of the periosteum on blood supply over the past decades, resulting that nearly 80% of the blood supply to the bone cortex is provided by the blood vessels of the periosteum (2).

In addition to the vascular system, the periosteum has demonstrated extraordinary osteogenic, chondrogenic and adipogenic potential under physical and chemical stimulation. The activated PDCs have been found to proliferate and differentiate into chondrocytes, osteoblasts and adipocytes. As a primary source of MSCs, PDCs have garnered significant scientific attention for regenerative approaches. Researchers have investigated the osteogenic and chondrogenic potential of PDCs in tissue engineering as well as the signals that regulate their differentiation (3). However, there is limited literature systematically summarizing the role of PDCs about their isolation, culture, characterization and potential in tissue engineering. Furthermore, the differences between PDCs and other common sources of MSCs remain controversial. Therefore, the study aims to provide an overview of current evidence, summarizing the isolation approaches, characterization of PDCs, and outlining their osteogenic, chondrogenic and adipogenic capacities. This information serves as a reference for their potential therapeutic applications in various clinical scenarios, with a particular focus on comparing them with other common sources of MSCs.

## Isolation and characterization of PDCs

### Isolation and culture of PDCs

The isolation and culture of PDCs for *in vitro* investigations provide valuable approaches to elucidate the characteristics of cell populations and the signals which regulate their differentiation. Gaining a clear understanding of the processes that enhance chondrogenic, osteogenic, and adipogenic differentiation in PDCs holds immense importance in the development of engineered tissues (4). This process in MSCs contains three main steps, including cell proliferation, migration and aggregation of cells, and differentiation with expression of growth factors and transcription (5). Accordingly, the culture conditions of PDCs that reproduced the processes above are necessary for the replication of this differentiation profile. Due to the thin and inaccessible nature of the periosteum, the isolation of PDCs has posed significant challenges in the past decades. Nevertheless, aided by the continuous advancement of techniques, more insight is acquired into the isolation and culture of PDCs.

**Figure 1:**
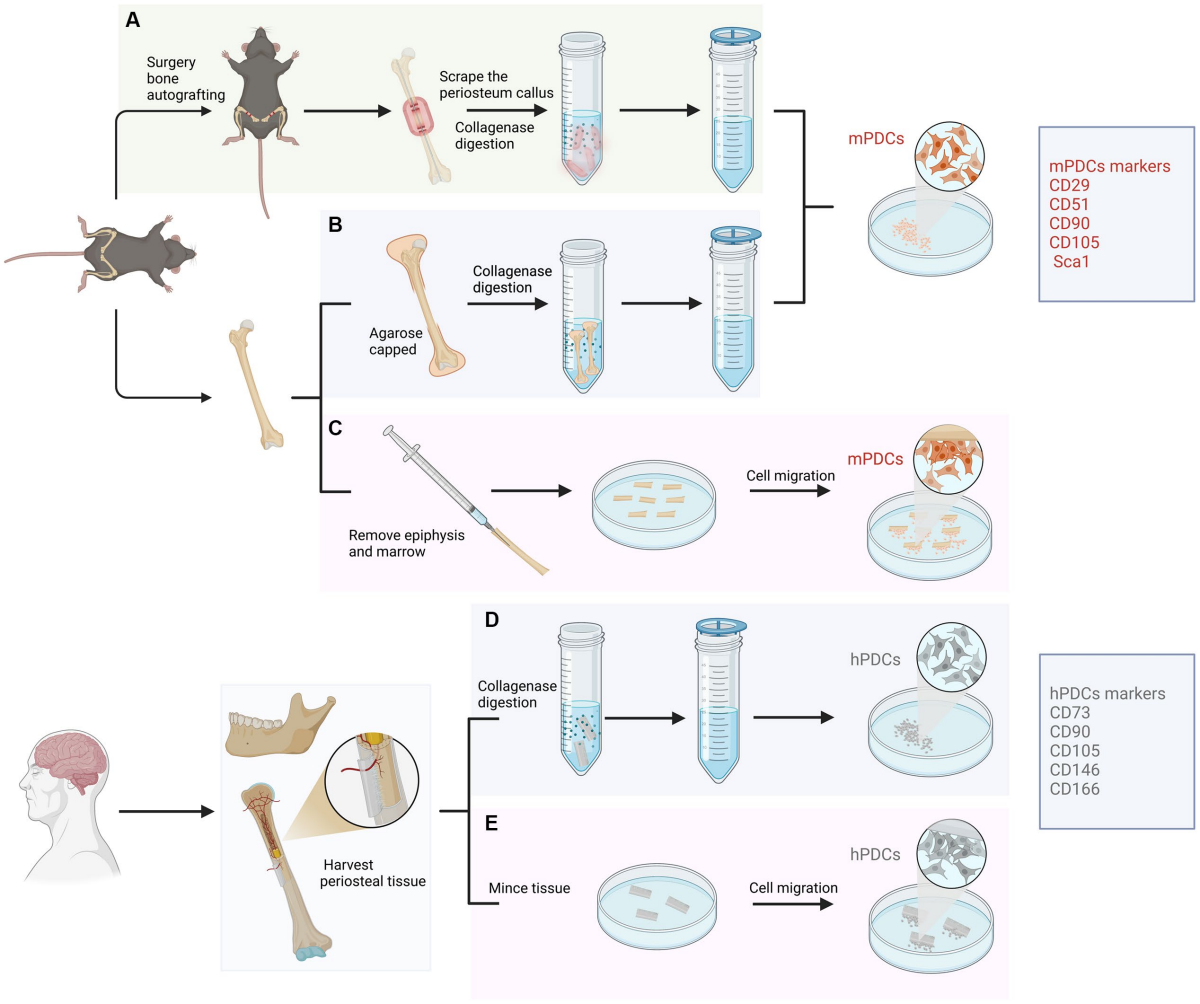
Isolation approaches and characterization of periosteum-derived cells (PDCs) from animal and human samples. For animals, PDCs are isolated from femur and tibia mainly using three approaches. **(A)** Isolated through an autograft resection and implantation surgery. Cells from the cambium layer proliferate, differentiate and migrate, forming a spongy callus. After several days, PDCs can be harvested from the callus through enzymatic digestion. **(B)** Isolated PDCs by bone capping with agarose. Femurs and tibias are dissected and epiphyses are capped with agarose. PDCs are liberated through enzymatic digestion and filtered through a cell strainer. **(C)** Isolated PDCs by placing long bones free of epiphyses, skeletal muscle and bone marrow. This approach allows PDCs to migrate from the periosteum to explant into the culture medium without any manipulation. For humans, PDCs can be obtained from the periosteum of long bones or the mandible through digesting in collagenase **(D)** or mincing the periosteal tissue **(E)**.

For human samples, PDCs were generally obtained by carefully removing the periosteum from the bone cortex through peeling or scraping, followed by explant culture or enzymatic digestion of the tissue (6). Similar approaches were employed for the isolation of PDCs in animals (7–9). There are generally three main methods utilized in the isolation of PDCs from mice. The first one involves harvesting PDCs by bone autografting through an autograft resection followed by implantation surgery (7). The second approach entails isolating PDCs by bone capping with agarose, which enables the direct harvest of PDCs that are within the periosteum (8). While the aforementioned methods have primarily been applied in mice, a recent protocol has been introduced placing long bones free of epiphyses, skeletal muscle and bone marrow to allow the proliferation and migration of PDCs (9). This method is more natural since it enables the migration of viable uncommitted and committed cells from the periosteum into the culture medium without any manipulation. In addition, the characterization of PDCs can be evaluated and compared with BMSCs which were obtained through direct bone marrow flushing. Other advantages of this approach include no need for callus formation or enzyme digestion. Isolated PDCs displayed high chondrogenic, osteogenic and adipogenic differentiation capacities *in vitro* and showed promising potentials *in vivo* ectopic transplantation experiments (Figure 1).

Various factors and methods have been demonstrated to influence the culture of PDCs (10). Initially, cortisol was considered an inhibitor of the proliferation of PDCs (11). Furthermore, the addition of dexamethasone and fetal bovine serum (FBS) during the early stages of differentiation has been reported to have a positive impact on the expression of osteocalcin and alkaline phosphatase (ALP) (12). Additionally, the incorporation of concentrated growth factor (CGF) or vascular endothelial growth factor (VEGF) into a basal culture medium has been shown to enhance both the proliferation and osteogenic potential of PDCs (13). Another study showed that the application of gelatin microspheres for cytokine release enhanced the cartilage formation ability of PDCs (14). In addition, PDCs were demonstrated to respond to mechanical force, leading to the highest expression of messenger RNA (mRNA) obtained under moderate (5–8%) anisotropic axial strain (15).

### Characterization of PDCs

The key features of isolated PDCs, assessed through cell surface markers, exhibit high comparability across studies. PDCs have been shown to display canonical mesenchymal markers such as CD29, CD51, CD105, CD90 and Sca1 in mice and CD73, CD90, CD146, CD105 and CD166 in humans (16, 17) (Figure 1). In contrast, markers associated with hematopoietic and endothelial lineages were absent (18–20). PDCs have been shown to express osteogenic markers such as osteopontin, osteocalcin, and collagen type I in an osteogenic environment, as well as chondrogenic markers like aggrecan and collagen type II under chondrogenic conditions (4). The expression of adipogenic markers such as AP2, leptin, lipoprotein lipase (LPL) and peroxisome proliferator-activated receptor have also been proved (21). Furthermore, Debnath et al. discovered periosteal stem cells (PSCs) in the calvarium and long bones of mice, which occupy the apex of a differentiation hierarchy (22). Both mouse and human PSCs possess the self-renewal potential and clonal multipotency in serial colony-forming units or ectopic transplantation experiments, which demonstrated their characterization as stem cells. One point that should be emphasized is that the number of PDCs may decrease with age. Cultured PDCs from younger donors consistently formed bone and cartilage, while cultured PDCs from older ones formed neither cartilage nor bone *in vivo* (23). However, aged PDC potential can be regenerated by transplanting Prx1+ PDCs at the bone defect site (24).

## Potential of PDCs in tissue formation

### Osteogenic capacity

The incidence of musculoskeletal disorders including osteoporosis, fractures and rheumatic diseases is rising rapidly because of the increase in life expectancy, the cell-based tissue engineering approach for the replacement of malfunctioning or defective tissues has garnered scientific attention. As a main source of MSCs for bone regeneration, the periosteum has demonstrated the ability to produce bone under optimal conditions (25–27). Evidence of the osteogenic capacity of PDCs in animal models was first described by Nakahara et al. (23). PDCs have been shown to secrete growth factors and extracellular matrix components that induce osteoblasts differentiation, which are responsible for bone formation. Cultured human PDCs presented bone formation capacity in a xenogeneic graft model under rat calvarial defects (28). Another study evaluated the osteogenic capacity through the synthesis of a tissue-engineered periosteum with cultured MSCs and acellular dermal matrix (29). Critical-sized bone defects were successfully healed after 6 weeks, which demonstrated the bone formation capacity of PDCs. Periosteal-derived macrophage-lineage cells are necessary for periosteum homeostasis and regeneration (30). The potential of human PDCs obtained from arthritic periosteal tissue has also been demonstrated (31). Furthermore, human jaw PDCs could be good candidates for tissue engineering to repair maxillofacial defects due to their potential bone formation capacity (32). It has been concluded that PDCs obtained from oral tissues could undertake the osteoblast lineage after nearly 2 months of culture in the absence of osteogenic induction (33).

**Figure 2:**
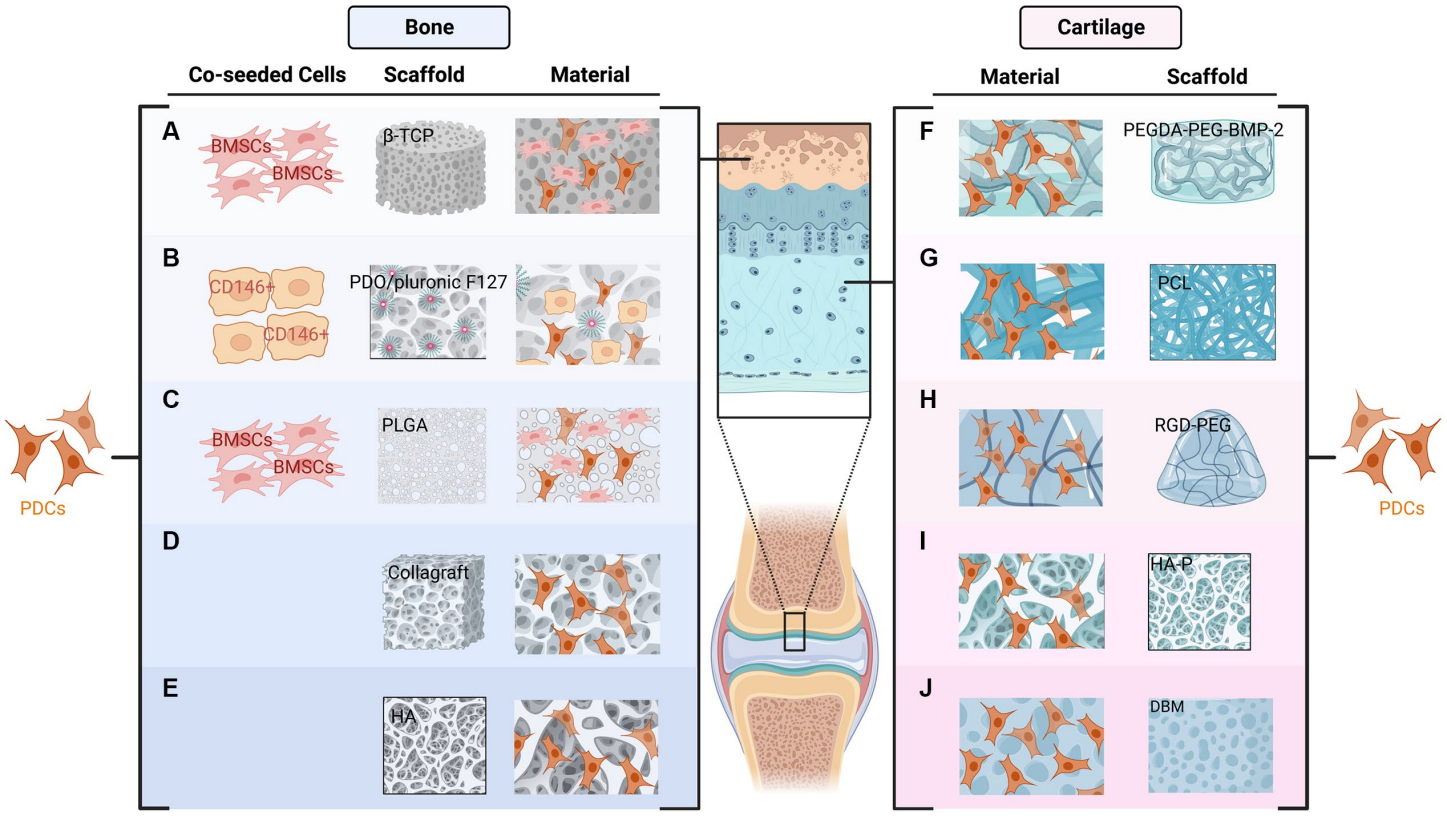
Combination of periosteum-derived cells (PDCs) and various types of scaffolds used in bone **(A–E)** and cartilage formation **(F–J)**. The New bone formation has been demonstrated after the co-cultured BMSC and PDCs combined with β-TCP **(A)** or PLGA **(C)** scaffolds. PDCs and PDO/Pluronic F127 scaffold with pre-seeded CD146 positive cells can be used to restore the bony defects of the maxillofacial region **(B)**. Collagraft **(D)** and HA **(E)** are other two scaffolds used for bone regeneration. Regarding cartilage formation, PDCs combined with PEGDA tethered with PEG and BMP2 can be applied to promote tendon-bone healing **(F)**. PDCs can infiltrate PCL scaffolds and form cartilage *in vitro* **(G)**. RGD-PEG hydrogels also support the proliferation and in vitro chondrogenesis of PDCs **(H)**. HA-P **(I)** and DBM **(J)** have also been used for cartilage formation.

Various scaffolds have been applied by researchers to improve osteogenic capacity in bone defects (34). For instance, human PDCs extracted from the mandible showed good bone formation capacity in three-dimensional collagen scaffolds (28). Poly (lactic-co-glycolic acid) (PLGA) scaffold has been applied in tissue engineering over the past decades. Improved bone quality was demonstrated after using this scaffold combined with PDCs and BMSCs (35). In addition, tissue-engineered bone using PDO/Pluronic F127 scaffolds and PDCs can be applied to repair maxillofacial defects (36). New bone formation was demonstrated after grafting in co-cultured BMSC and PDCs with β-TCP scaffolds (37). Collagraft was another widely used scaffold in the literature, PDCs formed mature bone *in vivo* after being transplanted on this type of scaffold (7, 20). Furthermore, Hydroxyapatite (HA) has also been applied in current studies accompanied by other materials such as tricalcium phosphate (TCP) and collagen (38) (Figure 2).

Several signaling pathways regulating the osteogenic differentiation of PDCs have been proposed in the literature. For example, as one earliest pathway up-regulated after a bone fracture, increased BMP expression was displayed in activated periosteum while reduced BMP expression negatively affects the proliferation, migration, and osteogenic differentiation of PDCs (39). BMP2 is essential in the fate decision of PDCs’ osteogenic differentiation since the bone formation capacity of PDCs can be improved by the exogenous addition of BMP2 (40). Concerning the Wnt and Notch pathways, the crosstalk between these two pathways is critical during the process of bone healing (41). The FGF pathway is another critical factor that participates in all stages including proliferation, migration and osteogenic differentiation of PDCs (42). In addition, activation of the Hedgehog pathway also significantly promoted the differentiation of PDCs *in vitro* and enhanced bone formation *in vivo* (43). Several of the above-mentioned pathways may be modulated by COX2, which plays a critical role in the activation of the PDCs (44, 45). Conversely, the PDGF pathway inhibits the osteogenic capacity of PDCs as the PDGF receptor beta may inhibit downstream target gene expression during BMP2-induced osteogenesis (46).

Accordingly, the findings above demonstrate the potential of PDCs for bone formation therapies in clinical scenarios. Further extensive clinical investigations should focus on the applications of PDCs in multiple complex settings.

### Chondrogenic capacity

As a highly specialized tissue helping synovial joints articulate under low friction, articular cartilage presented limited potential due to its avascular, aneural and lymphatic nature (47). Allografts may be applied for larger cartilage defects, but this method displayed the potential to trigger an immune response (48). Accordingly, there is growing interest in cell-based approaches used to repair or regenerate cartilage. Current studies have demonstrated the chondrogenic capacity of the periosteum (49–53). Using two different animal models, Mendes et al. indicated that stable cartilage can be formed when combined with PDCs, which can form ectopic cartilage (49). Another study concluded that the cartilage formation capacity of the periosteum for repairing cartilage defects can be enhanced with a pre-treatment of PDCs cultures with TGFβ3 *in vitro* (50). In addition, culture coatings comprising decellularized cartilage extracellular matrix drive robust and rapid chondrogenic differentiation of human PDCs (51). Although the numbers and potential of PDCs may decline with age, periosteum obtained from elderly samples still exhibits cartilage formation capacity and remains a promising source in cartilage regeneration (52). Furthermore, PSCs have been shown to have an impact not only on intramembranous bone formation but also on growth plate maintenance and prolonged longitudinal bone growth (53).

Several scaffolds were also used in cartilage regeneration. For example, Casper et al. demonstrated that PDCs can infiltrate poly-epsilon-caprolactone (PCL) nanofiber scaffolds in 6-month-old rabbits and subsequently form cartilage *in vitro* (54). PDCs combined with poly (ethylene glycol) diacrylate (PEGDA) tethered with PEG and BMP2 were introduced in another study to promote tendon-bone healing (55). In addition, human PDCs combined with enzymatically degradable poly (ethylene glycol) hydrogels functionalized with adhesion ligands to facilitate engineered cartilage have also been demonstrated (56). PDCs harvested from individual calves were able to produce cartilage *in vivo* after being implanted in nude mice combined with Hydroxyapatite-poly (HA-P) scaffold (57). In addition, demineralized bone matrix (DBM) has been used as a natural cell scaffold for cartilage formation and has displayed promising results (58) (Figure 2).

Various signaling pathways have been demonstrated to influence the chondrogenic capacity of PDCs, including FGF, BMP, TGF-beta, Wnt and Notch. The FGF pathway plays a key role during PDC activation regarding cartilage formation and maturation (59). In addition, the addition of TGF-beta 1 decreased the time of cartilage formation of PDCs and increased the amount of cartilage formed in the lower part of the culture (60). Sub-periosteal injection of TGF-beta can induce the proliferation and chondrogenic differentiation of PDCs (61). A previous study indicated that neochondrogenesis of PDCs was induced by BMP2 and the terminal differentiation in BMP2-induced cartilage formation was modulated by TGF beta 1 (62). Ryu et al. (57) indicated that Wnt-5a and Wnt-11 signaling have negative effects on type II collagen expression. The Notch pathway exhibits a similar effect as Wnt since the downregulation of Notch-2 expression has been observed during cartilage regeneration of PDCs (58). However, other signal pathways related to the regulation of cartilage formation are limited.

Accordingly, the chondrogenic capacity of PDCs has been demonstrated in current studies, and more in-depth researches are needed to elucidate the signaling pathways associated with them in terms of cartilage formation.

### Adipogenic capacity

Compared with studies investigating the osteogenic and chondrogenic capacity of PDCs, there is a paucity of research specifically focusing on their adipogenic potential. Adipogenic stimulation of PDCs resulted in the expression of the adipogenic marker genes aP2 and APM1 and the formation of lipid droplets (19). Another study also investigated the adipogenic differentiation of PDCs and observed the presence of lipid droplets on the cell monolayer (63). Both mitochondrial DNA contents and mitochondrial proteins were increased during the process of adipogenic differentiation of PDCs (21).

In contrast to the consensus regarding the osteogenic and chondrogenic capacity, studies have not led to an agreement on the adipogenic capacity of PDCs. Some studies suggest that fibroblasts and PDCs exhibit similar propensities for adipogenic differentiation when cultured in an adipose-inductive medium for 7 days (64). However, another study concluded that PDCs presented lower adipogenic capacity than BMSCs based on Nile red, which revealed the lower adipogenic potential of PDCs (65). Stich et al. (18) evaluated 51 different clonal cultures of human PDCs and found that only 52.9% could be stimulated to adipogenesis with histological and immunochemical staining. In contrast, 100 and 94.1% of the clonal cultures demonstrated the capacity for bone and cartilage formation, respectively (66). In addition, it seems that the adipogenic capacity of PDCs presents a high relationship with hyperglycemic conditions. The adipogenic differentiation of PDCs was altered by hyperglycemic conditions, and increased PPARγ expression in hyperglycemic conditions demonstrated the observed increase in differentiated adipocytes (67). However, there is currently a lack of relevant research investigating the signaling pathways associated with the adipogenic capacity of PDCs, as well as studies focusing on adipose tissue regeneration.

Accordingly, there were many challenges to drawing conclusions from the available literature in terms of the adipogenic capacity of PDCs due to the limited number of relevant studies and inconsistent conclusions. Further research is necessary to clarify the adipogenic capacity of PDCs and the underlying regulatory pathways.

### Other potential applications of PDCs

In addition to the osteogenic, chondrogenic and adipogenic capacity of PDCs, other potential applications for PDCs have also been demonstrated in current studies due to their stem cell attributes including self-renewal and multipotency. For example, researchers evaluated the influence of PDCs in ischemia–reperfusion-mediated renal fibrosis and concluded that PDCs presented stronger renoprotection and immunoregulation compared to BMSCs due to the promotion of PDCs for Treg differentiation through inhibiting the mTOR pathway (68). In addition, the potential application of the periosteum in combination with other tissues such as the epidermis and dermal papilla for tissue regeneration has also been demonstrated in previous studies (69). Recently, the antler-lineage periosteum also supplements the characteristics of the periosteum, which is capable of initiating ectopic organ formation upon transplantation and full mammalian organ regeneration when interacting with the covering skin (70). Moreover, the clinical investigations utilizing PDCs in maxillary sinus floor augmentation have shown lamellar bone formation within 3 months after transplantation, which provides a reliable basis for simultaneous or secondary insertion of dental implants and proves the therapeutic potential of PDCs (71). However, further trials are necessary to provide more comprehensive evidence for the efficacy of PDCs in different clinical settings.

## PDCs and other sources of MSCs

The potential differences between PDCs and other commonly used MSCs have been widely investigated in current studies. In general, PDCs demonstrated promising capacities when compared with other sources of MSCs. For example, Chen et al. reported that human PDCs exhibited higher mRNA of osteopontin, osteocalcin and BMP2 compared to BMSCs, and displayed stronger mineralization after osteogenic induction (72). Another study compared the proliferative capacities of MSCs from muscle, adipose tissue, periosteum and bone marrow, revealing that the periosteum yielded significantly higher numbers of MSCs, indicating their potential (73). Moreover, PDCs and BMSCs exhibited comparable potential for bone reconstruction in peri-implant defects, validating the utility of PDCs as an alternative MSC source in implant dentistry (74). However, these results should be interpreted cautiously as other studies have reported that while PDCs in the jaw bone exhibit osteogenic characteristics, they are less reliable than BMSCs in terms of bone formation capacity (75). Apart from BMSCs, other sources of MSCs have also been documented in the literature. For example, a recent study displayed the isolation and *in vitro* characterization of skeletal stem and progenitor cells (SSPC) (76). SSPC derived from metaphysis and endosteum exhibited the ability to differentiate into the osteogenic and adipogenic lineage, although their potential for cartilage formation was limited. Therefore, additional studies should focus on the applicability of PDCs in various contexts to confirm their comparative advantages with other conventional sources of MSCs.

## Conclusion

Overall, with the continuous advancement of techniques and tools, the characterization and regulation of PDCs can be comprehensively analyzed. As a subset of cells expressing canonical mesenchymal markers, PDCs offer great promise in the field of tissue engineering. PDCs exhibit osteogenic, chondrogenic and adipogenic potential, making them highly valuable for therapeutic applications. Moreover, PDCs have shown promising capabilities when compared to other sources of MSCs. Further studies should focus on exploring their clinical utility in various scenarios to confirm their comparative benefits over commonly used sources of MSCs.

## Author contributions

RC and BC contributed to the concept and research design and writing. KS, FG, and HP contributed to data collection. YC critically revised the manuscript. All authors contributed to the article and approved the submitted version.

## Funding

This research was funded by the Fujian Provincial Finance Department, grant number BPB-2022CYJ.

## Conflict of interest

The authors declare that the research was conducted in the absence of any commercial or financial relationships that could be construed as a potential conflict of interest.

## Publisher’s note

All claims expressed in this article are solely those of the authors and do not necessarily represent those of their affiliated organizations, or those of the publisher, the editors and the reviewers. Any product that may be evaluated in this article, or claim that may be made by its manufacturer, is not guaranteed or endorsed by the publisher.
